# Timing of indocyanine green injection prior to laparoscopic colorectal surgery for tumor localization: a prospective case series

**DOI:** 10.1007/s00464-020-07443-5

**Published:** 2020-02-18

**Authors:** Tetsuta Satoyoshi, Kenji Okita, Masayuki Ishii, Atsushi Hamabe, Akihiro Usui, Emi Akizuki, Koichi Okuya, Toshihiko Nishidate, Hiroo Yamano, Hiroshi Nakase, Ichiro Takemasa

**Affiliations:** 1 Department of Surgery, Surgical Oncology and Science, Sapporo Medical University, S1, W16, Chuo-ku, Sapporo, 060-8543 Japan; 2 Department of Gastroenterology and Hepatology, School of Medicine, Sapporo Medical University, S1, W16, Chuo-ku, Sapporo, 060-8543 Japan

**Keywords:** Colorectal cancer, Indocyanine green fluorescence imaging, Near infrared, Tumor location, Colonic marking, Laparoscopic surgery

## Abstract

**Background:**

Accurate identification of tumor sites during laparoscopic colorectal surgery helps to optimize oncological clearance. We aimed to assess the timing of the local injection preoperatively and clarify the usefulness and limitation of tumor site marking using indocyanine green (ICG) fluorescence imaging.

**Methods:**

Consecutive patients who underwent primary colorectal cancer surgery from September 2017 to January 2019 were included. Preoperatively, lower endoscopy was used to inject the ICG solution into the submucosal layer near the tumor. During laparoscopic surgery, ICG fluorescence marking as the tumor site marking was detected using a laparoscopic near-infrared camera system. The detection rate and factors associated with successful intraoperative ICG fluorescence visualization including the interval between local injection and surgery were evaluated.

**Results:**

One hundred sixty-five patients were enrolled. Using the laparoscopic near-infrared system, the intraoperative detection rates of ICG marking were 100% for ICG injection within 6 days preoperatively, 60% for injection between 7 and 9 days preoperatively, and 0% for injection earlier than 10 days preoperatively. There were no complications associated with ICG marking. Additionally, this method did not disturb the progress of the surgical procedure because injected ICG in the submucosal layer did not cause any tissue inflammation, and if ICG spilled into the serosa, it was invisible by white light.

**Conclusion:**

Advantages of ICG fluorescence tumor site marking were high visibility of infrared imaging during laparoscopic colorectal surgery and minimal adverse events of surgery. One of the most important findings regarding practical use was a rapid decrease in fluorescence marking visibility if one week passed from the time of ICG local injection.

Indocyanine green (ICG) has been used as an intraoperative navigation agent in several diseases [1, 2]. There have been some reports that tumor sites marking using ICG for colorectal cancer surgery has high visibility [3, 4], but the usefulness and features of ICG in clinical practice need to be clarified in detail in order for it to be widely used. Surgical approaches and the necessary resection margins in colorectal cancer are determined by the tumor sites, so accurate identification of tumor sites is important for maximizing oncological clearance and functional sparing.

To identify the tumor site during surgery, intraoperative colonoscopy may be necessary in some cases, but it can be resource-dependent and can cause colonic distension if not performed properly.

India ink tattooing has been conventionally used for tumor marking in colon cancer surgery. This technique was first described in 1976 by Ponsky and King [5]. Two studies reported that India ink tattooing was useful and safe for marking tumors in the colon [6, 7]. However, adverse effects caused by India ink tattooing have been observed; for instance, if India ink spreads widely throughout the peritoneal cavity, it can cause problems such as inflammation, focal peritonitis, inflammatory pseudotumors, abscesses, and adhesions [8–10]. Because India ink is a permanent tattooing method, it cannot be eliminated if it spills out of the serosa, which could be a major disadvantage when proceeding with laparoscopic surgery. Therefore, we need a novel agent characterized by lower rates of inflammatory reactions when in contact with the serosa, less visibility in white light, and lower surgical view interference rates.

In 1993, a study first described the use of ICG as a colonic marking agent instead of India ink, which was used with white light in open and laparoscopic surgery [11]. Then, ICG fluorescence imaging as the tumor site marking in near-infrared fluorescence was reported to be feasible and safe following local injection with endoscopy [3]; however, it has several unknowns. ICG is metabolized and excreted in vivo, but the timing of local ICG injection for marking purposes has been unclear when using near-infrared light. The interval from local ICG injection to surgery is also related to the number of endoscopies and the burden on the patient; therefore these factors should be considered over the long term. Another problem is that it is visualized with near-infrared light unlike India ink. The impact of these features on surgery is worth considering. If ICG spills into tissue, it is unclear whether it will disturb the surgical field or cause tissue inflammation like India ink, and this should be clarified. Therefore, in this study, we evaluated the detection rate of ICG fluorescence markings with near-infrared light in a relatively large number of patients and aimed to clarify the usefulness and limitation of it in laparoscopic colorectal surgery.

## Patients and methods

### Study population

From September 2017 to January 2019, consecutive patients scheduled to undergo laparoscopic colorectal resection in Sapporo Medical University Hospital were enrolled. Eligible patients were 20 years or older with histopathologically proven colorectal cancer, had an Eastern Cooperative Oncology Group performance status of 0–2, and provided informed consent. Patients were excluded if they had iodine hypersensitivity.

### Ethical statements

This study was approved by the Institutional Review Board of Sapporo Medical University, and patients who understood and agreed with the explanation of the research study were enrolled. Informed consent was obtained from all participants.

This clinical trial was registered in the University Hospital Medical Information Network (UMIN) Center (ID: UMIN000038982).

### Study procedure

**Fig. 1:**
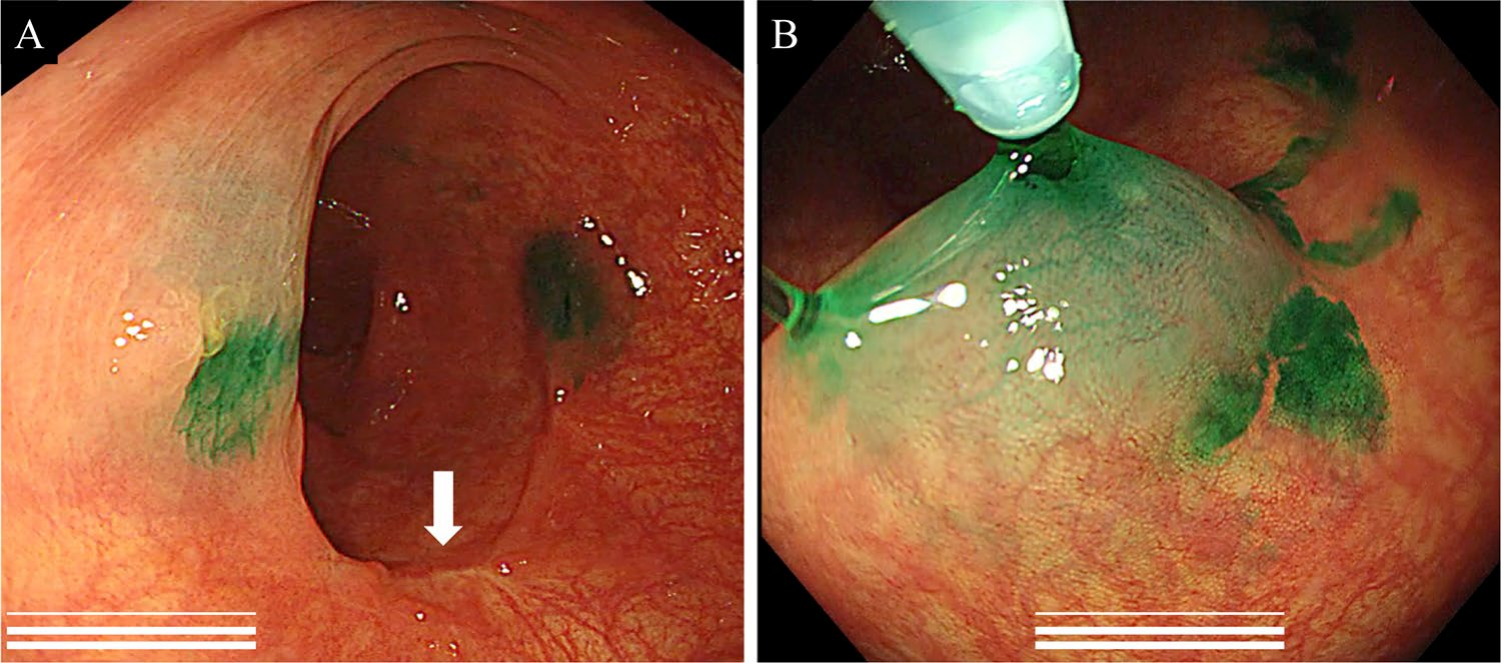
Preoperative indocyanine green (ICG) local administration. **A** The ICG solution is administrated into the submucosal layer at two points around the tumor using a colonoscope. The white arrow is the tumor site (this case was after endoscopic submucosal dissection). **B** Using a 26-gauge needle, 2 ml of normal saline solution was injected into the submucosal layer to form a submucosal elevation at two sites on the colonic wall opposite the tumor. Then 0.1 cc (ICG dose: 0.5 mg) of an ICG solution was injected into the elevated areas

In order to use ICG fluorescence imaging to detect the tumor site during surgery, we injected an ICG solution preoperatively into the submucosal layer at two points around the tumor using a colonoscope (Fig. 1A, B). The timing for the colonoscopy depended on the surgeon and the patient, and the change in the detection rate of ICG marking due to the interval between endoscopic marking and the operation was examined. During preoperative colonoscopy, a stock solution of ICG (Diagnogreen; Dai-Ichi Pharmaceuticals, Tokyo, Japan) was prepared by dissolving 25 mg of powdered ICG in 5 ml of sterilized water. Using a 26-gauge needle, 0.2 ml of a normal saline solution was injected into the submucosal layer to form submucosal elevation at two sites on the colonic wall opposite the tumor. Then 0.1 cc of ICG solution was injected into the elevated areas. The assistant pushed the dose accurately using a 1 ml syringe for tuberculin local injection. The local injection volume was set as the minimum volume that could be technically injected without problems. As a result of preliminary experiments, it was confirmed that fluorescence observation was possible with ICG doses between 0.125 and 2 mg. We set the dose at 0.5 mg, which was the median value and easily implicated in clinical practice. During the laparoscopic operation, ICG fluorescence marking was conducted using a laparoscopic near-infrared camera system (1588/1688 AIM laparoscope; Stryker, San Jose, CA, USA; DaVinci operation robot system; Intuitive Surgical, Mountain View, CA, USA). In this study, the detections were judged as positive if ICG fluorescence could be visualized as tumor site markings at the time of bowel resection. The detection rate was defined as the proportion of patients in whom the ICG fluorescence marking was observed during surgery by the operator.

### Primary and secondary endpoints

The primary endpoint was the intraoperative detection rate of ICG markings. Secondary endpoints were the appropriate timing of the ICG local injection, and the safety of the method was determined by evaluating the rates of complications and allergic events. The positive detection of ICG fluorescence was defined as whether ICG fluorescence could be used clinically as tumor site identification based on the operator’s judgement.

### Statistical analysis

The data are presented as mean and standard deviation or as median and range. Statistical analyses were performed using the statistical software program EZR (Easy R), which is based on R and R commander, and freely available (https://www.jichi.ac.jp/saitama-sct/SaitamaHP.files/statmed.html).

## Results

### Patient characteristics

One hundred sixty-five consecutive patients were enrolled in this study. All patients underwent laparoscopic surgery. The patient and tumor characteristics are presented in Table 1. All procedures were performed safely, with no complications, allergic events, or conversion to open surgery.

**Table 1:** Patient and tumor characteristics (*n* = 165)

Variable	Data
Age (years)	
Median (range)	67 (38–89)
Sex	
Male	89
Female	76
BMI (kg/m^2^)	
Median (range)	22.8 (15.8–35.4)
T.Bil	
Median (range)	0.6 (0.3–2.0)
Clinical stage (preoperative diagnosis)	
0	3
I	42
II	73
III	37
IV	10
Tumor location	
Right-sided colon	41
Left-sided colon	55
Rectum	69

*BMI* Body Mass Index, *T.Bil* total bilirubin

### Detection of ICG fluorescence marking

**Fig. 2:**
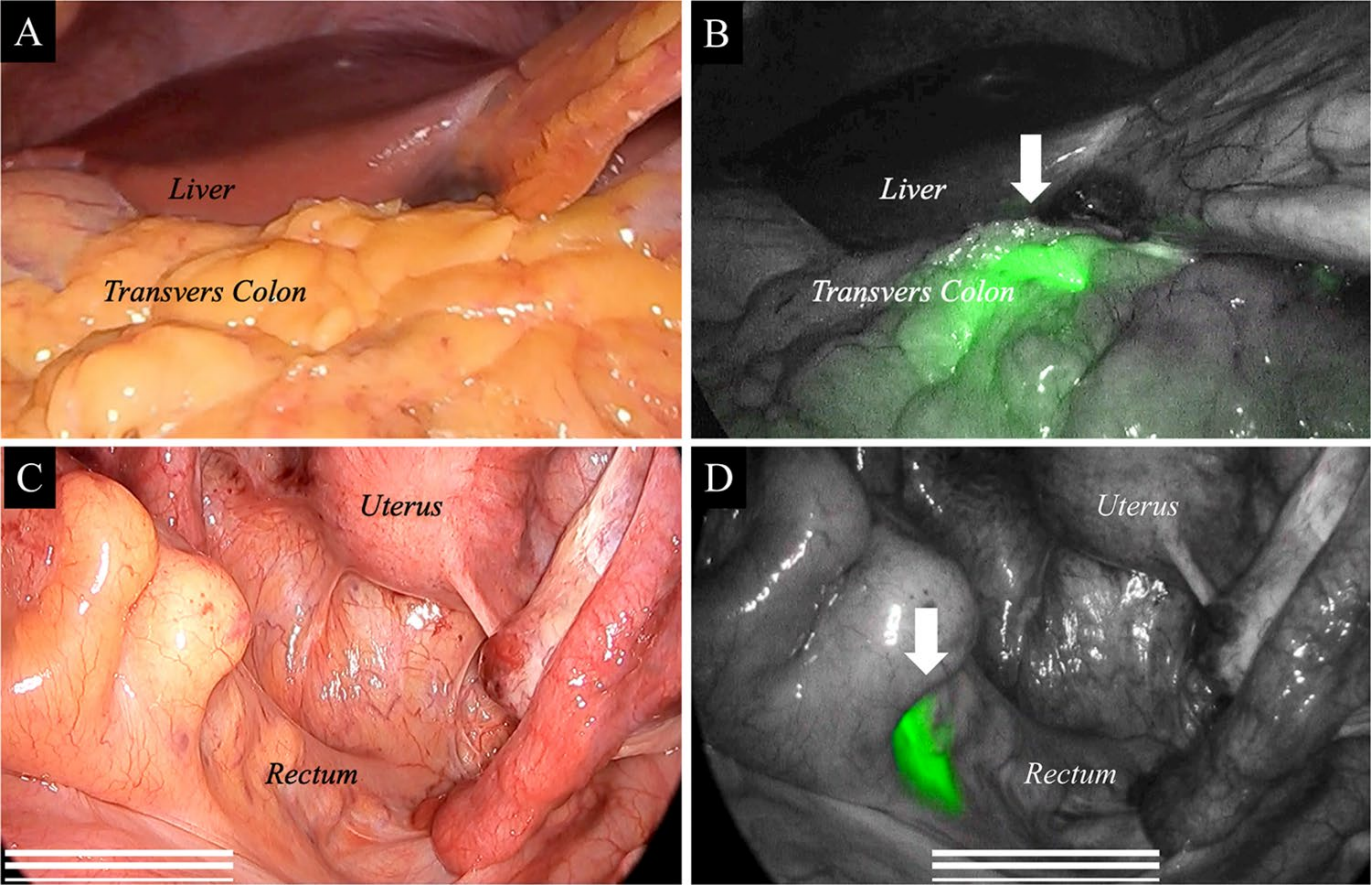
Indocyanine green (ICG) fluorescence marking is visualized during laparoscopic colorectal cancer surgery using a near-infrared camera. Transverse colon cancer (**A** white light view. **B** near-infrared view). Rectal cancer (**C** white light view. **D** near-infrared view). White arrow indicates the ICG fluorescence marking

**Fig. 3:**
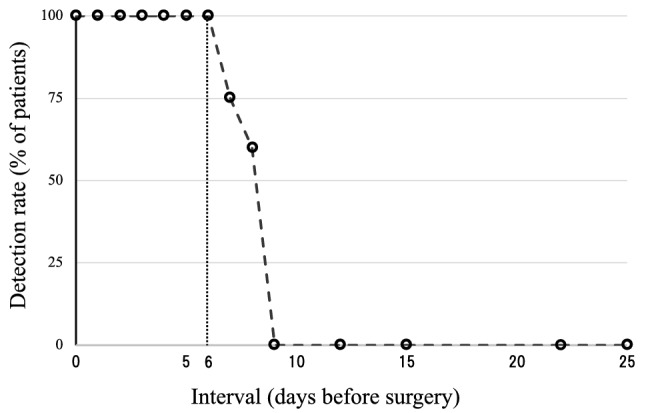
The relationship between the detection rate of indocyanine green (ICG) markings and the number of days between ICG injection and the day of surgery. The vertical axis indicates the detection rate. ICG fluorescence marking is detected in all patients within 6 days from the ICG injection. However, the detection rates rapidly decrease when more than 7 days has elapsed since the ICG injection

The markings using ICG fluorescence imaging were visualized with a near-infrared camera during laparoscopic surgery (Fig. 2A–D). The detection rates of intraoperative ICG fluorescence marking and the intervals between endoscopic marking and the subsequent operation are shown in Fig. 3. The detection rates rapidly decreased when more than 7 days has elapsed since the ICG injection. In all 141 patients who underwent ICG marking within 6 days, ICG fluorescence marking was detected with the near-infrared camera. ICG fluorescence marking was visualized in 6 of 10 patients (60%) who were marked between 7 and 9 days before surgery and in none of the four patients (0%) who were marked more than 10 days before surgery. The detection rates of ICG fluorescence were significantly different between these three groups (*p* < 0.0001, Fisher’s exact test).

### Complications

**Fig. 4:**
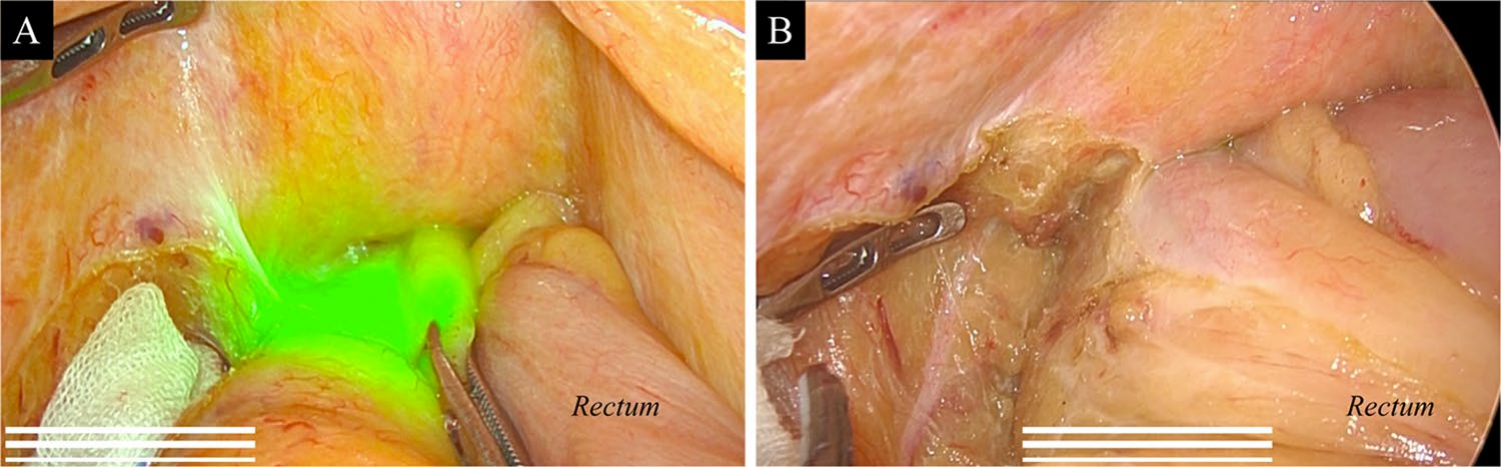
Mesorectal excision for rectal cancer during laparoscopic surgery. **A** The near-infrared overlay view. Indocyanine green (ICG) fluorescence marking aimed at the tumor site has spilled into the serosa and spread widely. Consequently, it obscures the separation boundary of mesorectal excision. **B** The same area was observed only in the white light view. ICG did not disturb the surgical field in the white light view. As a result, clear excision with nerve preservation was performed

**Fig. 5:**
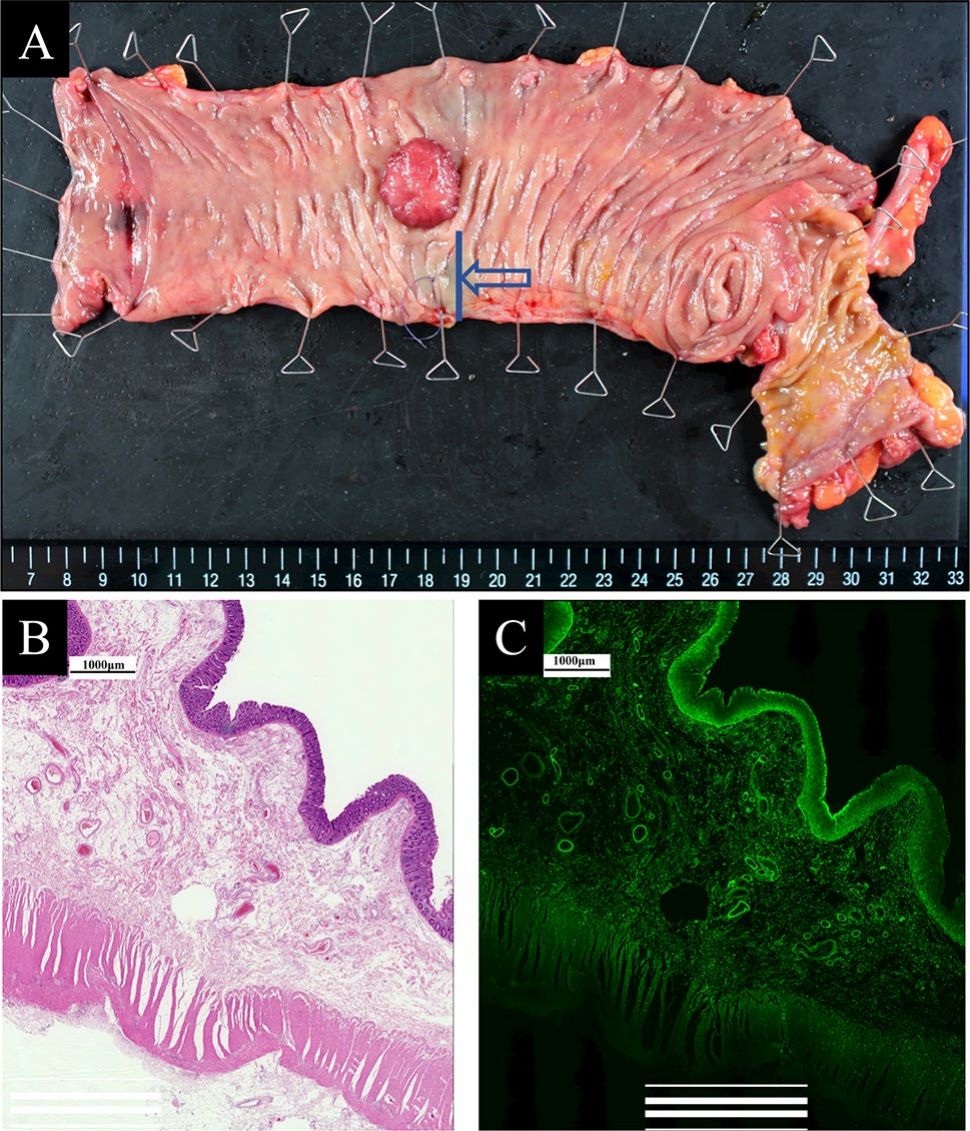
**A** Resected specimen of ascending colon cancer. The blue arrow indicates the point of indocyanine green (ICG) injection for tumor site marking. A pathological specimen is obtained at the blue line. **B** Microscopic view of the intestinal wall where ICG was injected. Hematoxylin and eosin staining showed the submucosal layer with mild interstitial swelling and a few inflammatory cell infiltrates. The serosa that has adhesions during surgery shows a large number of inflammatory cell infiltrates but no evidence of their spread from the submucosa. **C** ICG fluorescence microscopy of the same slice shows that the ICG molecules are distributed and fluoresced in the tissue of the submucosa, but these are not present outside the proper muscle layer

No patients experienced preoperative adverse events caused by local injection of ICG, such as fever or abdominal pain. Additionally, no patient had an allergic reaction to ICG. During intraoperative observation, ICG did not form an intraabdominal abscess in any case. In some cases, ICG fluorescence marking spilled into the serosa, spread widely, and obscured the separation boundary in the near-infrared view, but it did not disturb the surgical field in the white light view (Fig. 4A, B). In some cases, intraabdominal adhesions were observed, but their direct relationship with the ICG injection was not proven. Pathological examination in the case of a patient with peritoneal adhesion showed only mild tissue changes and no increase in the number of inflammatory cells in the submucosal layer into which ICG was locally injected; additionally, there was no spread of inflammation in the serosa where the adhesion occurred (Fig. 5A, B). ICG fluorescence microscopy of the same slice showed that the ICG molecules were distributed and fluoresced in the tissue of the submucosa and were not present outside the proper muscle layer (Fig. 5C). The same microscopic findings were observed in another case which did not cause intraabdominal adhesions.

## Discussion

This study showed that observation of ICG fluorescence marking with near-infrared light is a reliable method for tumor site marking if ICG is injected into the submucosal layer around the tumor within 6 days before laparoscopic colorectal surgery. Another finding was that the ICG substance maintained in the tissue of the submucosa and excitation wavelength penetrated the outer layer, so that ICG marking would not cause inflammatory cell migration outside the bowel wall. In addition, even if it has spilled into the serosa, it did not disturb the surgical field in the white light view. We clarified the usefulness of ICG fluorescence imaging for tumor site marking in laparoscopic colorectal surgery.

The methods of detecting the tumor site during surgery are limited. India ink tattooing was a common tumor site marking method. This process results in permanent markings, which is beneficial, but if it spreads widely outside the serosa, the accuracy of identifying the tumor location is lost. Additionally, the black-stained surgical field makes the operation difficult to complete.

In terms of surgical principle, complete mesocolic excision (CME) for colon cancer and total mesorectal excision (TME) for rectal cancer surgery have been shown to significantly improve outcomes [12–16]. An accurate marking identification using ICG fluorescence imaging of the tumor site during surgery would be useful in both CME and TME. Therefore, ICG marking was suggested to be one of the techniques to improve the prognosis of colorectal cancer surgery.

In a previous study of ICG use for tumor site marking, 1 ml (ICG dose: 12.5 mg) of ICG suspension was injected into the mucosal layer. The positivity of staining and interval between ICG marking and surgery were assessed in detail by observation in the visible light spectrum [4]. In our study, the ICG markings were visualized by near-infrared light; thus, their detection was possible with an injection of only 0.1 ml (ICG dose: 0.5 mg) of ICG suspension. The near-infrared fluorescence of ICG has a wavelength of 800–1000 nm and good biopermeability [17]. Therefore, it is considered that even if a small amount of ICG suspension is injected into the submucosal region, the fluorescence of ICG passes through the intestinal wall and can be detected with high reproducibility from outside the serosa. The advantage of using a small injection volume is that it can reduce the risk of expanding the ICG fluorescence area and prevent misidentification of the tumor site. It also reduces the risk of leakage into the peritoneum, which can cause peritonitis and intestinal adhesions.

In a previous study of ICG marking in laparoscopic colorectal surgery, fluorescence induced by near-infrared light enabled surgeons to clearly and accurately identify the tumor location in all 24 patients who were injected with ICG within 96 h before surgery [3]. The details of the correlation between visualization of ICG fluorescence and the timing of the preoperative injection of ICG before surgery are unclear in cases where ICG markings are observed using near-infrared light in laparoscopic colorectal cancer. We assessed tumor site marking in 165 patients who underwent laparoscopic colorectal cancer surgery. Our study results suggest that ICG marking injection should be performed within at least 6 days before surgery. When the injections were performed more than 7 days before surgery, there were cases in which ICG fluorescence could not be visualized, and this incidence increased according with the intervals, presumably because the injected ICG suspension was cleared from the submucosal layer. However, the detailed mechanisms whereby the ICG molecules in the intestinal wall are metabolized and excreted are not yet known. In our study, liver function was not associated with the detection rate of the ICG fluorescence marking. As for the method of the local injection of ICG, the method using the endoscope like in the present study and local injection from outside of the serosa laparoscopically have been reported [18]. The endoscopic approach was considered suitable for marking the tumor site because the endoscope can detect early cancer and scar post local resection. Local injection of ICG into the submucosal tissue near the tumor resulted in fluorescence of the injection site. At the same time, fluorescence associated with lymph nodes and draining lymphatic flow was also observed. One study that assessed fluorescence in lymph node imaging reported that ICG could be safely injected into the peritumoral subserosal area and then subsequently demonstrated lymphatic drainage in those with colon cancer [19]. It is necessary to further examine how the simultaneous observation of ICG markings and ICG fluorescence of lymph nodes affect colorectal cancer resection. In the future we may investigate the pathological mechanisms and their association with fluorescence in lymph nodes.

This study has several limitations. First, adequate local injection into the submucosal layer required an endoscopist with high-level skills. If this technique fails, the accuracy of the tumor site is reduced and microperforation of the bowel can occur, subsequently causing inflammatory reactions. Second, in this study, only the detection rate of the ICG fluorescence marking was evaluated; ICG fluorescence imaging could differ depending on the local injection location. In particular, in some case of rectal cancer, diffuse expanding fluorescence signals of the marking were observed. Therefore, more consideration will be needed with regard to tumor marking location and the quality of tumor identification.

In conclusion, we clarified the usefulness and features of the tumor site marking method using ICG fluorescence imaging in laparoscopic colorectal cancer surgery. ICG fluorescence marking with near-infrared light is a reliable method for tumor site marking if ICG is injected into the submucosal layer around the tumor within 6 days before laparoscopic colorectal surgery.
